# The Influence of CLSM Magnification on the Measured Roughness of Differently Prepared Dental Materials

**DOI:** 10.3390/ma17235954

**Published:** 2024-12-05

**Authors:** Martin Rosentritt, Anne Schmutzler, Sebastian Hahnel, Laura Kurzendorfer-Brose

**Affiliations:** Department of Prosthetic Dentistry, UKR University Hospital Regensburg, 93042 Regensburg, Germanysebastian.hahnel@ukr.de (S.H.);

**Keywords:** surface roughness, R_a_/S_a_, R_z_/S_z_, confocal laser scanning microscopy, CLSM, magnification, dental materials

## Abstract

This in vitro study investigated how varying magnifications (5×, 10×, 20×, and 50×) using a confocal laser scanning microscope (CLSM) influence the measured surface roughness parameters, R_a_/S_a_ and R_z_/S_z_, of various materials with two surface treatments. Cylindrical specimens (d ≈ 8 mm, h ≈ 3 mm, *n* = 10) from titanium, zirconia, glass-ceramic, denture base material, and composite underwent diamond treatment (80 μm; wet) and polishing (#4000; wet; Tegramin-25, Struers, G). The surface roughness parameters (R_a_/S_a_, R_z_/S_z_) were measured with a CLSM (VK-100, Keyence, J) at 5×, 10×, 20×, and 50× magnifications. Line roughness (R_a_/R_z_) was measured along a 1000 μm distance in three parallel lines, while area roughness (S_a_/S_z_) was evaluated over a 2500 μm × 1900 μm area. The statistical analysis included ANOVA, the Bonferroni post hoc test, and Pearson correlation (SPSS 29, IBM, USA; α = 0.05). R_a_/S_a_ and R_z_/S_z_ showed significant differences (*p* ≤ 0.001, ANOVA) across magnifications, with values decreasing as magnification increased, highest at 5× and lowest at 50×. Titanium, zirconia, and glass-ceramic showed significant measured roughness values from 5× to 50×. Denture base material and composite had lower measured roughness values, especially after polishing. Line and area roughness varied significantly, indicating that magnification affects measured values. Standardizing magnifications is essential to ensure comparability between studies. A 50× magnification captures more detailed profile information while masking larger defects.

## 1. Introduction

Surface roughness is an important factor in assessing dental materials because it influences bacterial adhesion, biofilm formation, gloss, color stability, biocompatibility [1,2,3,4] and strength [5]. Smooth surfaces of dental restorations enhance aesthetics and reduce biofilm adhesion. Rough surfaces can promote the growth of microorganism on denture materials [3], potentially leading to oral diseases such as denture stomatitis or caries [2,6]. Additionally, the susceptibility of dental materials to discoloration is influenced by their surface roughness [7,8]. Studies have shown that rough surfaces are more susceptible to color changes, which also reduces the color stability of the material [9]. In addition, the roughness depth appears to be an important factor in reducing the flexural strength of dental materials [5,10].

Surface roughness is typically quantified using roughness parameters such as R_a_/S_a_ (arithmetic mean roughness/arithmetic mean surface roughness) and R_z_/S_z_ (average roughness depth/maximum height of the surface) [4,11]. R_a_ and R_z_ are two-dimensional parameters that measure line roughness. S_a_ and S_z_ are three-dimensional parameters that measure the surface roughness over an area. R_a_ and S_a_ provide an overview of the average surface roughness and effectively summarize the surface topography. In contrast, R_z_ and S_z_ capture peak-to-valley variations and reflect the more extreme surface features. The use of widely established roughness metrics facilitates a comparison between different studies [10,11,12,13,14,15]. Together, these parameters provide a wide analytical scope for the evaluation of surfaces.

The confocal laser scanning microscope (CLSM) is an optical, non-contact method for determining surface roughness. In CLSM, the surface is scanned with a laser beam, which is an alternative to contact profilometry, which is based on the physical interaction between the surface and a diamond tip. In confocal microscopy, light from a laser is passed through the objective of a conventional light microscope to excite a sample in a narrow focal plane. To selectively illuminate the specimen and filter out diffuse signals during CLSM measurements, the light waves are focused through a narrow aperture [14]. The specimen absorbs this light energy and emits it again in longer wavelengths, which are then captured and converted into an image [14,16]. A CLSM thus produces “optical” sections of the surface. These successive sections are then converted into digital images and a topographic map. To calculate the roughness parameter, these digital maps are described and analyzed using an algorithm.

The results of CLSM measurements can vary depending on the surface treatments applied and the specific dental materials used [17]. Therefore, the effects of different dental materials and surface treatments on the roughness parameters in general applications must be taken into account. A CLSM usually provides different measured roughness values than a contact profilometer for measurements of a single and identical surface, especially for surface area roughness parameters as opposed to line roughness parameters [10,14,17]. A surface can be described as a superposition of numerous wavelengths, whereby the transition from the particularly long-wave form components to the waviness and short-wave roughness components of the surface takes place smoothly. Roughness, shape, and waviness are therefore not sharply defined characteristics. The roughness is separated from the waviness with the help of frequency filters, which essentially decide which surface features are defined as waviness and which as roughness. The settings selected for the CLSM, such as the cut-off wavelength (λ_s_, λ_c_), therefore have an influence on the measured roughness values and the resolution [14,18]. The precision of measurements using a CLSM depends on the careful selection of key factors, such as scan distance, light intensity, vertical resolution, and lens type [19,20]. For reliable results, Buajarern J., Kang C., and Kim J. recommend maintaining a light intensity of no less than 90% [19].

The CLSM is widely employed for measuring both two-dimensional and three-dimensional roughness parameters [19,21]. The advantage of three-dimensional roughness measurement is that it is not subject to the sensitivity of the measuring position and therefore provides more reliable results—especially for inhomogeneous and defective surfaces. Additionally, the CLSM is commonly utilized for detailed 3D image analysis [19,21,22].

Studies often evaluate devices with varying measurement principles, including tactile profilometry, CLSM, scanning electron microscopy, or phase-shifting interferometry, to analyze roughness parameters [15,19,20]. Variations in the results are largely attributed to the fundamental differences in these devices’ operational principles. In profilometry, the surface is mechanically analyzed, whereby the depth of entry into the microstructure is limited by the size of the diamond tip or stylus [20]. In contrast, a CLSM performs non-invasive surface scanning, enabling the capture of fine details and deeper surface valleys [20,23].

Comparative analyses of dental materials such as titanium, zirconia, PEEK, and ceramics have been conducted using optical measurement devices, considering various surface treatments and both line and area roughness parameters [10,11,15,17,22]. However, no studies to date have specifically investigated the impact of varying CLSM magnification levels on roughness parameter measurements.

A particularly important parameter in this context is the selected lens magnification. There is currently a lack of studies comparing the roughness parameters R_a_/S_a_ and R_z_/S_z_ at different magnifications using a CLSM. It is important to understand how different magnifications affect the measurement of roughness values, as the accuracy and reproducibility of these measurements can depend significantly on the parameters chosen.

Assessing the measured roughness of dental materials is crucial for clinical applications, as it directly impacts adhesion, aesthetics, and durability. Variations in roughness measurements caused by magnification levels, material types, and surface treatments highlight the need to thoroughly analyze these influencing factors. This is particularly important when evaluating and comparing results across multiple studies to ensure consistency and accuracy.

The aim of this in vitro study was to investigate how different CLSM magnifications influence the measured surface roughness parameters R_a_/S_a_ and R_z_/S_z_ on different dental materials and surface treatments. This study hypothesizes that CLSM magnification influences the measured surface roughness values of the parameters R_a_/S_a_ and R_z_/S_z_ for different dental materials and surface treatments.

## 2. Materials and Methods

### 2.1. Specimen Preparation

Cylindrical specimens (d ≈ 8 mm, h ≈ 3 mm) were prepared from various dental materials: titanium (Grade 4), zirconia (Cercon HT, Dentsply, Bensheim, Germany), glass-ceramic (Empress, Ivoclar-Vivadent, Schaan, Liechtenstein), denture base material (Palapress, Kulzer, Hanau, Germany), and composite (Grandio, Voco, Cuxhaven, Germany). The materials were selected because they reflect the broad spectrum of dental applications (e.g., titanium: implants; zirconia and glass-ceramic: fixed partial dentures; denture base material: dental prostheses; and composite: filling) and are also used in similar forms and varieties in technical applications.

Each specimen underwent two types of surface treatments: diamond treatment (80 μm; wet) and polishing (silicon carbide foil with a grit of 4000; Buehler, Düsseldorf, Germany; wet; Tegramin-25, Struers, Willich, Germany).

### 2.2. Measurement Device

The surface roughness parameters R_a_/S_a_ and R_z_/S_z_ were measured using a confocal laser scanning microscope according to ISO 25178-2:2019 (surface/area roughness parameters for parameter S_a_/S_z_) [24] and ISO 21920-2:2022-12 (line roughness for parameters R_a_/R_z_) [25] (range = 7 mm, z-resolution = 0.005 μm, x-resolution = 0.01 μm, repeatability = 0.02–0.05 μm, ND-filter 100%, VK-100, Keyence, Osaka, Japan). Measurements were conducted at various magnifications (5×, 10×, 20×, and 50×) for each specimen (*n* = 10 per material and surface treatment). The line roughness parameters (R_a_/R_z_) were determined using three parallel lines, each measuring 1000 μm, while the area roughness parameters (S_a_/S_z_) were assessed over a square area of 2500 μm × 1900 μm. Cut-off wavelengths of λ_s_ = 0.8 μm and λ_c_ = 0.08 mm were applied during measurements. The size of the measuring area (length × width) was adjusted according to the selected magnification level: 5× magnification, ~2450 μm × 1800 μm; 10× magnification, ~1450 μm × 1085 μm; 20× magnification, ~705 μm × 525 μm; and 50× magnification, ~285 μm × 210 μm.

### 2.3. Statistical Analysis

An analysis of variance (one-way ANOVA) was carried out to check whether there were statistically significant differences between more than two groups. ANOVA was used to compare the mean values of the individual parameters of the respective groups. A pairwise comparison of means (Bonferroni post hoc test) was performed to investigate significant differences between the means of the individual groups. The Pearson’s correlation coefficient based on covariance was applied to assess the strength and direction of the relationship between the continuous variables examined. The level of significance was set to α = 0.05 in all tests. All statistics were performed with SPSS 29, IBM, Armonk, NY, USA.

## 3. Results

### 3.1. Magnifications—Overview

**Figure 1:**
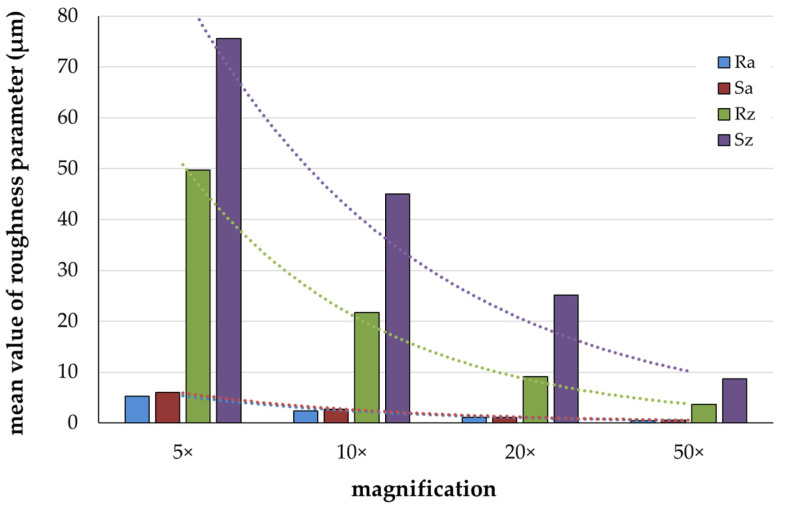
Trend of the mean roughness parameters R_a_/S_a_ and R_z_/S_z_ (μm) in relation to magnification (overview of all materials). The summarized data show the trend of reduced roughness with increasing magnification.

Figure 1 presents the development of the mean values for the roughness parameters R_a_/S_a_ and R_z_/S_z_ across magnifications ranging from 5× to 50×. With increasing magnification, the measured roughness values of R_a_/S_a_ and R_z_/S_z_ converged. At 5× magnification, the measurements showed the highest values. A reduction by a factor of 2 was observed at 10× magnification compared to 5×. An increase in magnification to 20× led to a further decrease by a factor of 3 (S_z_) to 5 (R_a_/S_a_ and R_z_) relative to 5×. The most pronounced reduction occurred at 50× magnification, where the values decreased by a factor of 9 (S_z_) to 14 (R_z_) in relation to S_z_ and R_z_ at 5× magnification. The differentiation between the R_a_ and S_a_ values at 5× and 50× magnification was less pronounced than the differentiation observed for the R_z_ and S_z_ values. 

The one-way ANOVA revealed significant differences between the groups (*p* ≤ 0.001). The Bonferroni post hoc test showed significant roughness parameters (*p* ≤ 0.036) when comparing the magnifications, but no differences (*p* = 1.000) between the magnifications of 20× and 50× for any roughness parameter. Additionally, a significant Pearson correlation was found between the magnification and the surface parameters (*p* ≤ 0.001).

**Figure 2:**
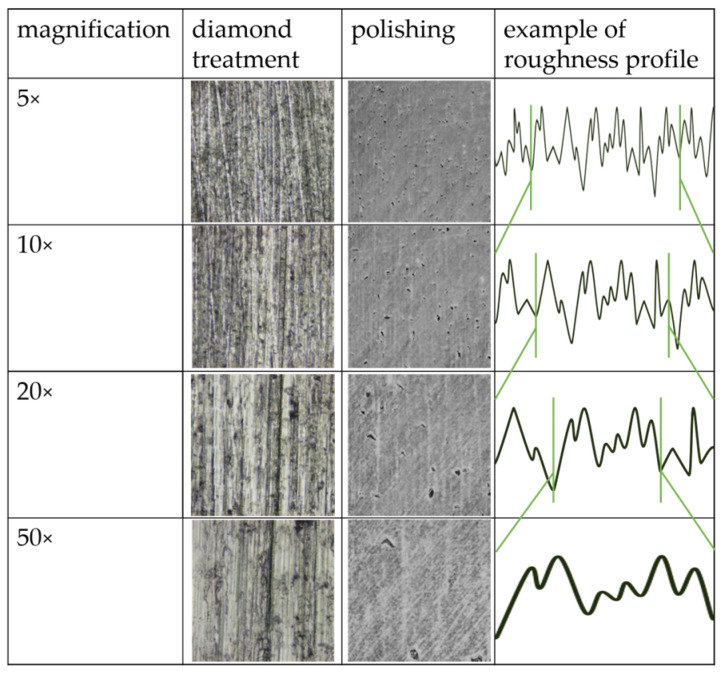
Examples of titanium surfaces with diamond treatment and polishing from 5× to 50× magnification, with an exaggerated example of the roughness profile to illustrate the effect of magnification.

Figure 2 presents a representative selection of a titanium specimen with diamond treatment and polishing at magnifications ranging from 5× to 50×. A comparison of the images reveals that the section of the specimen surface diminishes in size with increasing magnification, thereby facilitating a more discernible observation of its structural characteristics. Additionally, the effect of magnification on roughness measurement is highlighted through the exemplary depiction of the surface roughness profile.

### 3.2. Materials

**Figure 3:**
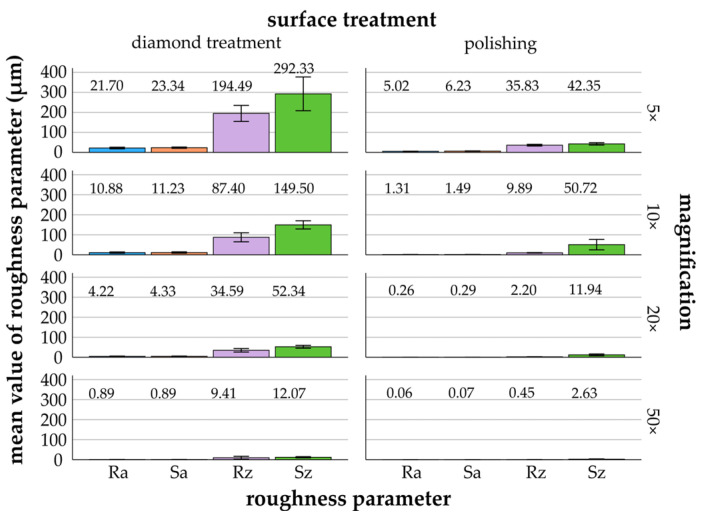
Titanium: mean values and standard deviations of R_a_/S_a_ and R_z_/S_z_ (μm) in relation to different surface treatments and magnifications (5× to 50×)—titanium (mean values are shown above the bars).

**Figure 4:**
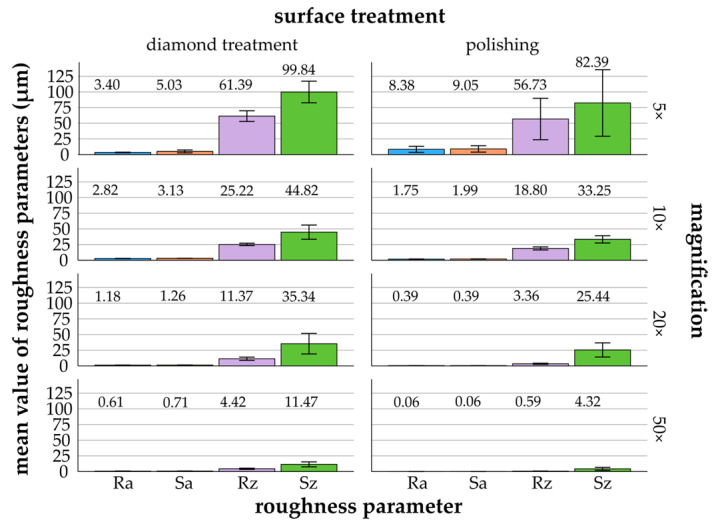
Zirconia: mean values and standard deviations of R_a_/S_a_ and R_z_/S_z_ (μm) in relation to different surface treatments and magnifications (5× to 50×)—zirconia (mean values are shown above the bars).

**Figure 5:**
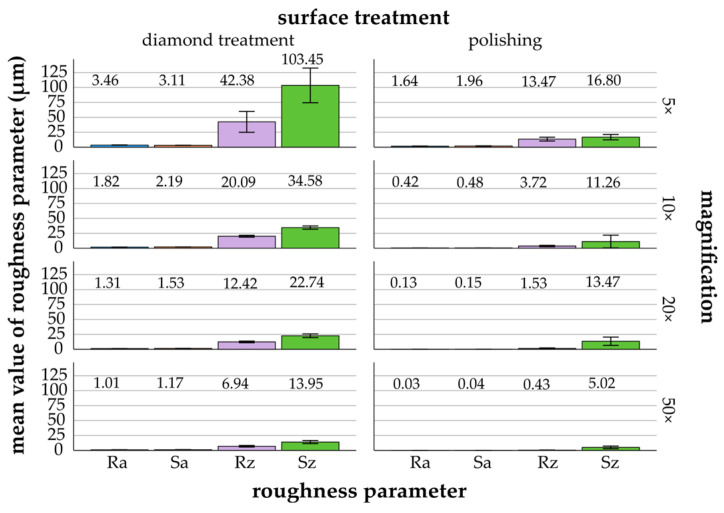
Glass-ceramic: mean values and standard deviations of R_a_/S_a_ and R_z_/S_z_ (μm) in relation to different surface treatments and magnifications (5× to 50×)—glass-ceramic (mean values are shown above the bars).

**Figure 6:**
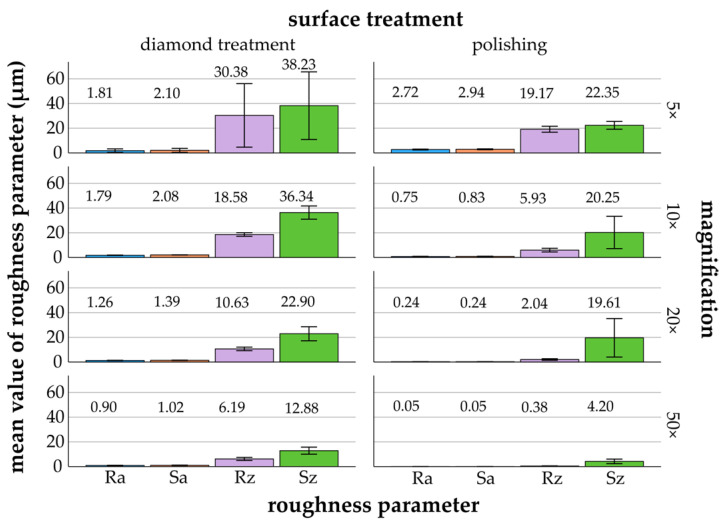
Denture base material: mean values and standard deviations of R_a_/S_a_ and R_z_/S_z_ (μm) in relation to different surface treatments and magnifications (5× to 50×)—denture base material (mean values are shown above the bars).

**Figure 7:**
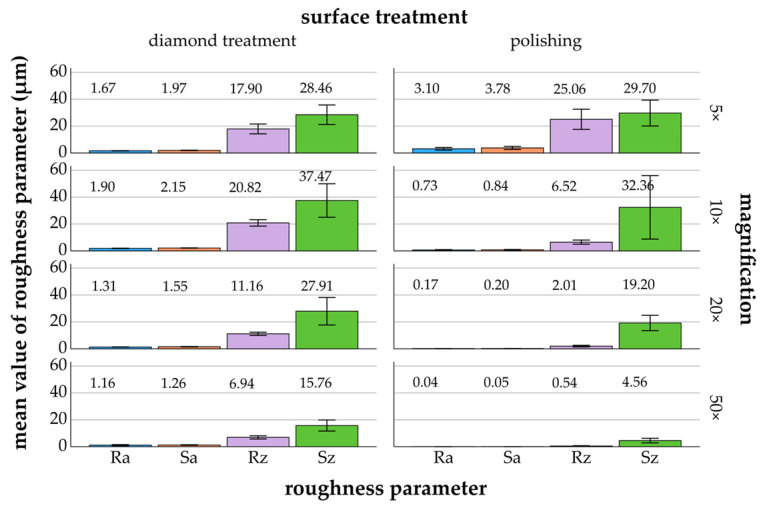
Composite: mean values and standard deviations of R_a_/S_a_ and R_z_/S_z_ (μm) in relation to different surface treatments and magnifications (5× to 50×)—composite (mean values are shown above the bars).

Figure 3, Figure 4, Figure 5, Figure 6 and Figure 7 provide details of the data for each material, including surface treatments (diamond treatment and polishing) and magnifications (5×, 10×, 20×, and 50×). The surface roughness parameters R_a_/S_a_ and R_z_/S_z_ showed significant differences between the different materials and surface treatments (*p* ≤ 0.022, ANOVA). Among the roughness parameters, S_z_ consistently exhibited the highest values across all materials and surface treatments and thus differed clearly from the other parameters. The differences between the measured S_z_ values and the R_a_/S_a_ and R_z_ parameters decreased with increasing magnification and fine surface treatment. Titanium, zirconia, glass-ceramics, and composite showed more significant differences after diamond treatment than after polishing. In contrast, the denture base material showed greater significant differences following polishing than diamond treatment.

#### 3.2.1. Titanium

In general, the mean values of R_a_/S_a_ and R_z_/S_z_ for titanium measured by the CLSM were highest at 5× (21.70 ± 4.04 μm/23.34 ± 3.12 μm and 194.49 ± 39.88 μm/292.33 ± 84.29 μm), 10× (10.88 ± 3.26 μm/11.23 ± 3.45 μm and 87.40 ± 22.65 μm/149.50 ± 20.47 μm), and 20× (4.22 ± 1.61 μm/4.33 ± 1.59 μm and 34.59 ± 8.84 μm/52.34 ± 7.13 μm) magnification with diamond treatment (Figure 3). The measured roughness values decreased and converged as the magnification increased. Specifically, the mean R_z_ values decreased by a factor of 20 from 5× magnification (194.49 ± 39.88 μm) to 50× magnification (9.41 ± 7.43 μm). Similarly, S_z_ decreased by a factor of 24 from 292.33 ± 84.29 μm at 5× magnification to 12.07 ± 2.95 μm at 50× magnification. After polishing, S_z_ showed a higher mean value at 10× (50.72 ± 26.05 μm) compared to 5× (42.35 ± 5.85 μm).

*Diamond treatment:* The Bonferroni post hoc test revealed significant differences (*p* ≤ 0.021) between magnifications for R_a_/S_a_ and R_z_/S_z_, except between 20× and 50× for R_a_ and R_z_/S_z_.

*Polishing*: Significant differences were observed between the magnifications for all four roughness parameters (*p* ≤ 0.001, Bonferroni), except between 20× and 50×, and between 5× and 10× for S_z_.

#### 3.2.2. Zirconia

Zirconia exhibited the highest mean values for R_a_/S_a_ and R_z_/S_z_ at magnifications of 5× ((8.38 ± 4.86 μm/9.05 ± 5.13 μm) and (56.73 ± 33.10 μm/82.39 ± 53.07 μm)), 10× ((1.75 ± 0.20 μm/1.99 ± 0.10 μm) and (18.80 ± 2.44 μm/33.25 ± 5.77 μm)), and 20× ((0.39 ± 0.11 μm/0.39 ± 0.09 μm) and (3.36 ± 0.94 μm/25.44 ± 11.27 μm)) for polishing compared to the other materials (Figure 4). Specifically, the mean values decreased with increasing magnification by a factor of 6 (R_a_) to 14 (R_z_) for diamond treatment and by a factor of 19 (S_z_) to 149 (S_a_) for polishing.

*Diamond treatment*: The Bonferroni test (*p* ≤ 0.010) indicated significant differences for the roughness parameters R_a_ and R_z_ at all magnifications. Partial significance was found for S_a_ and S_z_, with some exceptions: 10× and 20× for S_z_ and 20× and 50× for S_a_.

*Polishing*: Compared to the other materials, the magnifications for R_a_/S_a_ and R_z_/S_z_ showed the lowest significant differences (*p* ≤ 0.002; Bonferroni). For all roughness parameters, significant differences were found between 5× and the other magnifications (10×, 20×, and 50×). However, no significant differences were observed between 10×, 20×, and 50× for R_a_/S_a_ and R_z_/S_z_.

#### 3.2.3. Glass-Ceramic

The mean values decreased with increasing magnification for diamond treatment, by a factor of 3 (S_a_) to 7 (S_z_), and for polishing, by a factor of 3 (S_z_) to 50 (R_a_/S_a_) (Figure 5). The mean values became increasingly similar with decreasing magnification. Glass-ceramic showed the lowest mean values for R_a_/S_a_ and R_z_/S_z_ at 5× magnification, with 1.64 ± 0.27 μm/1.96 ± 0.29 μm and 13.47 ± 3.19 μm/16.80 ± 4.52 μm; at 10× magnification, with 0.42 ± 0.11 μm/0.48 ± 0.10 μm and 3.72 ± 1.13 μm/11.26 ± 10.69 μm; and at 20× magnification, with 0.13 ± 0.04 μm/0.15 ± 0.04 μm and 1.53 ± 0.77 μm/13.47 ± 6.94 μm, after polishing. S_z_ exhibited lower mean values at 10× (11.26 ± 10.69 μm) compared to 20× (13.47 ± 6.94 μm).

*Diamond treatment*: Significant differences were observed for R_a_/S_a_ at all magnifications (*p* ≤ 0.001, Bonferroni). Significant differences were also noted for R_z_/S_z_ (*p* ≤ 0.021, Bonferroni), except between 10× and 20×, and between 20× and 50× for both parameters.

*Polishing*: R_a_/S_a_ and R_z_ generally showed significant differences (*p* ≤ 0.047, Bonferroni), except for values between 20× and 50×. For S_z_, no significant differences were found between most magnifications.

#### 3.2.4. Denture Base Material

Denture base material exhibited the lowest mean values for diamond treatment across all parameters (R_a_/S_a_ and R_z_/S_z_) for magnifications of 10× (1.79 ± 0.15 μm/2.08 ± 0.09 μm and 18.58 ± 1.45 μm/36.34 ± 5.36 μm), 20× (1.26 ± 0.16 μm/1.39 ± 0.16 μm and 10.63 ± 1.42 μm/22.90 ± 5.69 μm), and 50× (0.90 ± 0.16 μm/1.02 ± 0.17 μm and 6.19 ± 1.23 μm/12.88 ± 2.85 μm) (Figure 6). The mean values constantly decreased with increasing magnification for both surface treatments. For diamond treatment, the mean value decreased by a factor of 2 (R_a_/S_a_) to 5 (R_z_). When polished, the mean values decreased by a factor of 5 (S_z_) to 59 (S_a_). Regarding S_z_ in polishing, the mean values showed a minor difference between 10× (20.25 ± 13.10 μm) and 20× (19.61 ± 15.58 μm).

*Diamond treatment*: The Bonferroni post hoc test (*p* ≤ 0.036) revealed few significant differences. R_a_ showed no significant differences between magnifications (*p* = 1.000). S_a_ and R_z_/S_z_ also generally showed no significant differences.

*Polishing*: Significant differences (*p* ≤ 0.012, Bonferroni) were found for R_a_/S_a_ and R_z_, except between 20× and 50×. For S_z_, no significant differences were found between most magnifications (*p* = 1.000).

#### 3.2.5. Composite

For the diamond-treated composite, the roughness parameters R_a_/S_a_ and R_z_/S_z_ exhibited lower mean values at 5× magnification, 28.46 ± 7.26 μm/1.97 ± 0.16 μm and 17.90 ± 3.66 μm/1.67 ± 0.13 μm, compared to 10× magnification, 37.47 ± 12.46 μm/2.15 ± 0.11 μm and 20.82 ± 2.36 μm/1.90 ± 0.15 μm (Figure 7). After polishing, a similar trend was observed for the roughness parameter S_z_, with mean values of 29.70 ± 9.62 μm at 5× magnification and 32.36 ± 23.59 μm at 10× magnification.

*Diamond treatment*: Significant differences were found for R_a_/S_a_ and R_z_ between magnifications (*p* ≤ 0.029, Bonferroni), with exceptions between 5× and 10× for R_a_/S_a_ and R_z_, and between 20× and 50× for R_a_. For S_z_, less significant differences were found between most magnifications.

*Polishing*: The Bonferroni test (*p* ≤ 0.028) revealed significant differences between magnifications for R_a_/S_a_ and R_z_, without significant differences between 10×, 20×, and 50×. No significant differences were found for S_z_ between most magnifications.

## 4. Discussion

The hypothesis that CLSM magnification influences the measured surface roughness results of parameters R_a_/S_a_ and R_z_/S_z_ across different dental materials and surface treatments could be confirmed. Based on the results, it is important to emphasize the careful selection of magnification levels in CLSM because these levels significantly influence the surface roughness parameters for different dental materials and surface treatments.

### 4.1. Magnifications—Overview

This study demonstrated that the surface roughness parameters measured with CLSM decreased with increasing magnification. R_z_/S_z_ decreased more with diamond treatment and R_a_/S_a_ with polishing. The lowest roughness results were measured at 50× magnification, where the measurements displayed the lowest standard deviations. At 20× and 50× magnification, the measured roughness values of the materials were equalized and the influence of the materials was reduced. These results suggest that higher magnification allows for more detailed and consistent roughness measurements. The highest measured roughness values were determined for all parameters at 5× magnification. The 5× and 10× magnifications demonstrate the material differences more clearly: specifically, titanium and glass-ceramic showed stronger material influence, presumably due to higher gloss and reflection [26]. Both roughness values R_z_ and S_z_ are sensitive to scratches and irregularities [27], which directly increase the measured roughness values. This could explain the higher measured roughness values observed for the rough surface treatments, especially at lower magnifications, as the selected magnification images a larger surface area (Figure 2).

In the current study, the different magnifications gave different measurement results for the different dental restorative materials. However, significant differences were found between the roughness parameters for all materials and surface treatments. S_z_ consistently displayed the highest measured roughness values, which underlines its sensitivity in detecting surface irregularities [27,28]. The convergence of S_z_ values with other parameters at higher magnifications indicates that higher magnification increases measurement accuracy, particularly for finer surface treatments. At 50× magnification, however, the measurements only capture a smaller section of the surface. This may limit the representativeness of the results. Therefore, the number of measurement repetitions at 50× magnification should be increased.

### 4.2. Materials

The measurement results seem to depend more on the composition of the materials than on the very strong differences in the properties. Titanium (modulus 200 GPa, Vickers hardness HV10) and glass-ceramics (80–120 GPa, HV 7) show a similar reduction in roughness due to polishing. Resin-based materials such as composite (20 GPa, HV 2) and denture base material (2–3 GPa, HV 1–2) generally show a similar performance with less influence from polishing. The deviating behavior is shown by zirconia (200 GPa, HV 10–12).

#### 4.2.1. Titanium

For titanium, the measured roughness values decreased with increasing magnification. Especially after diamond treatment, a drastic reduction from 5× to 50× was observed. This suggests that roughness may be overestimated at lower magnifications due to the greater influence of surface irregularities. The consistent decrease in measured roughness values with increasing magnification underlines the importance of using higher magnifications, such as 20× or 50×, for accurate surface characterization of rough titanium surfaces. Polished titanium surfaces are less affected by changes in magnification, so that lower magnifications are suitable for their evaluation. The surface treatments show clear differences between the measured roughness values. For the surface treatments, the magnification 5× and 10× show clear differences in the measured roughness values.

The use of a single line roughness parameter may limit the significance of the surface characterization [10]. Since R_a_ inverts all depths below a central line and treats them as heights, this can obscure a detailed understanding of the true properties of the surface. Cha et al. [28] measured the surface roughness parameters S_a_ and S_z_ on titanium threads at 450× magnification using CLSM. They concluded that the S_a_ values of the surfaces in all groups were in the “moderately rough” category, as previously defined [29]. However, an increase in the mean value and, above all, the standard deviation of S_z_ was observed. Despite the relatively unchanged S_a_ values, the increased roughness values measured for S_z_ indicate surface damage caused by the in-instrumentation methods [28]. A similar study, in which titanium surfaces were examined with the CLSM, showed comparable behavior when measuring surface roughness [30]. The roughness parameters R_a_ and S_a_ yielded similar values with low standard deviations, which indicates consistent results. However, S_z_ recorded the highest values for both fine and rough surfaces [30].

#### 4.2.2. Zirconia

Zirconia showed the highest measured roughness values at lower magnifications after polishing. The values decreased significantly with increasing magnification. Compared to titanium, the effect of polishing was less pronounced and the differences between the two treatments remained small at all magnifications. The significant reduction in roughness at higher magnifications indicates that lower magnifications can exaggerate the roughness of zirconia surfaces. In a study with 1000× magnification, R_a_ and R_z_ were measured on zirconia surfaces with different treatments [31]. At this higher magnification, the differences between the treated surfaces are clearer than at the lower magnifications used in this study [31].

#### 4.2.3. Glass-Ceramic

Glass-ceramic materials showed a similar trend of decreasing roughness with increasing magnification, especially after diamond treatment. The highest mean roughness was observed for both surface treatments at 5× magnification, indicating that this material may appear rougher at lower magnifications. The convergence of measured roughness values at magnifications of 10× and higher indicates that finer surface details become more prominent, which increases the accuracy of characterization. The consistent measured roughness values observed with polishing at different magnifications suggest that effective surface characterization is possible even at low magnifications. In a similar study, comparable behavior was observed in glass-ceramic specimens with different surface treatments, where the R_a_ values were determined at 50× magnification with a CLSM [32]. Despite the different surface treatments, R_a_ showed minimal differences in this magnification [32].

#### 4.2.4. Denture Base Material

For both surface treatments, decreasing measured roughness values were observed with increasing magnification. The results show that for diamond treatment, roughness may be overestimated at lower magnifications. This indicates that higher magnifications should be used to obtain more reliable surface measurements. For polished surfaces, a lower magnification, such as 10×, may be suitable, as the measured roughness values vary only slightly from this magnification, with the exception of S_z_. In a study comparing the different manufacturing methods of denture base materials without surface treatment, R_a_ was measured using a CLSM at 5× magnification [22]. In line with the results of the current study, the R_a_ values were low at this magnification.

#### 4.2.5. Composite

The composites showed little differentiation between surface treatments at 5× magnification, especially for S_z_. Polishing may exaggerate the roughness at low magnifications. In addition, the measured roughness values (R_z_/S_z_) increased at 10× magnification, suggesting that lower magnifications may exaggerate surface irregularities. Therefore, higher magnifications should be used for a more accurate evaluation and differentiation of various surface treatments. A similar behavior of R_a_ and R_z_ was observed in a study in which surfaces with different surface treatments were analyzed at 50× magnification [26].

### 4.3. Magnification Impact on Measured Roughness Values

Magnification is the ability of a microscope to display the image of an object at a larger scale than its actual size. This means that the microscope’s field of view of the microscope (object field) that is used for evaluation (and thus the section of the object that is reproduced in the final image) depends on the selected magnification. The measured values can be influenced accordingly. Higher magnification levels allow for a higher resolution, which generally provides a clearer and more detailed view of the microstructure of the surface. The contrast is determined by the minimum distance between two points (pixel distance). This means that even small irregularities can be resolved and recognized, thus improving the roughness measurement. Furthermore, the roughness measurements depend heavily on the scale at which the surface is scanned. Higher magnifications reduce the field of view, meaning the microscope captures a smaller area with more detail. This approach is ideal for analyzing roughness at the micro- or nano-scales as it highlights small features. However, conversely, lower magnifications provide a wider field of view. Smaller and finer roughness features may be smoothed out or overlooked. The result may be a lower roughness value.

Among the materials, titanium and zirconia exhibited the most prominent variability in measured roughness values as a function of the individual magnifications, especially after diamond treatment. The influence of the type of material on measured roughness values was more pronounced at lower magnifications. The results are certainly due to the large field of object but also due to the distinctive material properties compared to other materials, such as the high elasticity modulus of around 200 GPa.

In addition, zirconia and composite showed only slight differences in the measured roughness between the surface treatments. The results may certainly be influenced by the high hardness of the zirconia and also by the type and size of the filler of the composite. Furthermore, the differences in surface roughness between different materials illustrate the complexity involved in the CLSM measurement. The materials showed considerable differences in roughness at different magnifications, especially after diamond treatment. However, at higher magnifications, the measured roughness values for these materials converged, suggesting that higher magnifications may reduce material-specific variations that are more pronounced at lower magnifications.

The dependence of the measured surface roughness on the magnification highlights the importance of selecting suitable magnification levels for CLSM analyses. Lower magnifications may overestimate roughness by including more pronounced surface irregularities. Higher magnifications may offer a more detailed surface analysis by capturing finer structures. Therefore, the magnification should be chosen carefully and depending on the individual objectives to ensure accurate surface roughness measurements. Materials such as titanium and zirconia are particularly affected by lower magnifications, highlighting the need for material-specific settings. The standardization of protocols for different materials and surface treatments could improve the reproducibility and comparability of different studies. For example, using a 5× magnification may result in different roughness measurements compared to a 50× magnification. A 5× magnification covers a larger surface area than a 20× or 50× magnification, allowing larger topographical features such as sporadic height variations to be captured. In contrast, a 50× magnification offers a more detailed analysis of a smaller surface area [19] that is less affected by sporadic height variations, resulting in lower measured roughness values. The measuring field depends on the magnification of the objective as well as on the numerical aperture, the working distance, and the diameter of the focal point. The rule of thumb for selecting the measuring field should be five to ten times the scale of the coarsest structure of interest. In the range of maximum measured roughness (S_z_) between 30 µm and 300 µm, a measuring field size between 150 and 300 µm^2^ or between 1500 and 3000 µm^2^ should be selected depending on the material. If necessary, higher magnifications should be employed.

The differences that arise when measuring different materials are certainly due to the surface characteristics on the one hand. Reflective surfaces such as titanium should behave differently from matte materials such as composites. The different light refractions of roughened or polished samples should also be reflected in the measurement.

A frequency filter splits off the components of the surface that lie below or above the so-called cut-off wavelength (profile-based evaluation) or the nesting index (area-based evaluation). This filtering (e.g., λ_s_ and λ_c_ filters) is not carried out sharply at one wavelength, but by continuously attenuating the frequency components to be excluded. The results may also be due to the fact that confocal systems use filter algorithms to reduce noise or that larger surface ripples and waves are no longer visible at very high magnification. When determining surface characteristics, the choice of cut-off wavelength determines whether components are evaluated as waviness or roughness. The smaller the cut-off wavelength, the more rough parts of the surface are included in the profile. As a consequence, the roughness values are smaller. The same surface will show different measured values at different cut-off wavelengths.

### 4.4. Limitations and Future Research

The results of this study are certainly limited by the selection of dental materials and surface treatments. Although the selected materials are clinically representative, they do not cover all possible materials and procedures used in dentistry. The following applies to the quality of the measurement: the higher the magnification, the shorter the focal length. The inclusion and comparison of multiple CLSM devices in future studies could also improve robustness. Therefore, the magnification should be matched to both the material and the surface treatment, and a uniform magnification within studies should ensure reliable comparisons. For materials like titanium and zirconia, where the roughness varies greatly depending on the magnification, higher magnifications are crucial to avoid overestimating. In contrast, for materials with less variability at higher magnifications such as glass-ceramics and composites, magnifications of 20× or higher are advisable to capture finer surface details.

## 5. Conclusions

In summary, this study emphasizes the influence of magnification on the measurement of the surface roughness of dental materials and shows the importance of selecting appropriate magnification settings. The influence of material type and surface treatment on roughness measurements, particularly at low magnifications, should not be underestimated.

A lower magnification is recommended for the roughness parameters R_a_/S_a_, as these parameters are less affected at reduced magnification levels. R_z_/S_z_ may require higher magnification for more accurate measurements, as they remain more stable at higher magnifications. Consistent magnification is crucial when evaluating different roughness parameters.

For rougher surfaces resulting from diamond treatment, a higher but moderate magnification is required so that the roughness is not overestimated. Polishing can be assessed at lower magnifications. The observed variations in measured roughness values between different materials despite uniform surface treatment indicate the need for material-specific magnification settings.

## Data Availability

The data presented in this article are available on request from the corresponding author. The data are not publicly available due to privacy.
